# Updated plant hardiness zones for Canada and assessment of change over time

**DOI:** 10.1038/s41598-025-00931-5

**Published:** 2025-07-02

**Authors:** Daniel W. McKenney, John H. Pedlar, Kevin Lawrence, Kaitlin DeBoer, Heather MacDonald

**Affiliations:** Natural Resources Canada, Canadian Forest Service, Great Lakes Forestry Centre, 1219 Queen Street East, Sault Ste. Marie, ON Canada

**Keywords:** Plant ecology, Climate change

## Abstract

Plant hardiness systems have been developed for various regions around the world to help ensure that cultivated plants are grown at locations where suitable climate conditions prevail. In Canada, a multivariate plant hardiness index was developed in the 1960s that incorporates several temperature- and precipitation-related variables, as well as snow depth and wind speed. In the United States, the plant hardiness system involves averaging annual extreme minimum temperatures over a period of interest, with values subsequently classified into hardiness zones. Here we report on efforts to update hardiness zone maps for Canada using both the Canadian and US approaches and using climate data for the 1991–2020 period. The two hardiness systems produced generally similar spatial patterns in plant hardiness across Canada, including high index values in southern and coastal regions and low index values in northern and high-elevation areas. Detailed comparisons to previous hardiness maps indicated that, since 1961–1990, zone values have increased by between half a zone and two full zones across the country, with the largest increases occurring in western and northwestern Canada. For the multivariate Canadian hardiness system, a change attribution analysis indicated that three temperature-related variables were primarily responsible for driving the observed changes in the plant hardiness zones. The new maps are available at http://planthardiness.gc.ca.

**Supplementary Information:**

The online version contains supplementary material available at 10.1038/s41598-025-00931-5.

## Introduction

Climate has long been identified as a key driver of plant distribution and abundance^1^. As such, climate is thought to act as a coarse filter on the suite of species that can grow at a given location^2^. Plant hardiness systems, which typically involve a combination of mapped hardiness zones and plant hardiness ratings, have been developed to ensure that cultivated plants are grown under suitable climate conditions^3^. However, given recent and projected changes in climate^4^, ongoing updates to hardiness systems are critical for maintaining accurate hardiness zone maps.

Numerous plant hardiness systems have been developed around the world. In Canada, a multivariate regression approach that modelled plant survival as a function of seven climate variables was developed in the 1960s^5–7^. Updates to this zonation, using modern mapping techniques and revised climate data, were subsequently generated for the 1961–1990 and 1981–2010 periods^8,9^. In the United States, a hardiness zone map based on annual extreme minimum temperature was developed by the Department of Agriculture (USDA) in the 1960s^10^, with several updates issued since^11,12^. A Canadian version of the USDA extreme minimum temperature hardiness zone map has also been developed^9,13^. Furthermore, an extreme heat map, which delineates zones based on extreme maximum temperatures, has been developed for the United States^14^. Globally, plant hardiness systems—often based on extreme minimum temperatures—have been developed for various countries/regions^15–17^. Typically, long-term average climate conditions are used in plant hardiness calculations, with the assumption that short-term weather events (that can damage or kill plants), are captured by these longer-term summaries.

Species-specific distribution models offer an alternative to generalized plant hardiness zones^18^. In theory, such products would allow customized maps of the climatic range for each plant species/variety of interest under current and/or future climate. Despite an explosion in distribution modelling methods^19^ and associated datasets^20–22^, there remains a demand for the general direction that zonation approaches provide. As well as their primary function of guiding horticultural planting decisions, hardiness zone maps have been used for ecologically informed landscape design^23^, phytosanitary risk analyses^24^, and various forestry applications^25,26^.

Canada’s climate has changed significantly over the past century, with projections for continued change in the coming century^4^. Specifically, average temperatures have increased by approximately 1–3 °C across the country since the middle of last century, with larger shifts associated with temperature extremes^27^. Precipitation has increased by approximately 0–30% across Canada over the same period^27^. These changes have significant implications for plant growth and survival across the country, hence the need to regularly update plant hardiness zone maps to reflect these ongoing shifts. Several studies have reported hardiness zone shifts—typically toward warmer conditions—in relation to recent climate change^11,9,15,16^.

Here we provide updated plant hardiness maps for Canada, using both the Canadian and USDA approaches, along with climate data for the 1991–2020 period. We describe the development and assessment of the requisite spatial climate models and associated maps. We further provide detailed comparisons with past models (i.e., 1961–1990 and 1981–2010), including an exercise to identify which of the various climate variables used in the Canadian model are driving changes in the plant hardiness index across the country.

## Methods and data

### Plant hardiness models

As described previously^5–8^, the Canadian plant hardiness index was developed by modelling the survival of a set of 174 plants and cultivars at 108 locations across Canada. The resulting multiple regression model incorporates a suite of 7 climate variables as defined below:

1 $$Y={\text{ }} - {\text{67}}.{\text{62 }}+{\text{ 1}}.{\text{734}}{X_{\text{1}}}+{\text{ }}0.{\text{1868}}{X_{\text{2}}}+{\text{ 69}}.{\text{77}}{X_{\text{3}}}+{\text{ 1}}.{\text{256}}{X_{\text{4}}}+{\text{ }}0.00{\text{6119}}{X_{\text{5}}}+{\text{ 22}}.{\text{37}}{X_{\text{6}}} - {\text{ }}0.0{\text{1832}}{X_{\text{7}}}$$

where, *Y* = estimated index of survival (or suitability); *X*_1_ = monthly mean daily minimum temperature (°C) of the coldest month; *X*_2_ = mean frost free period above 0 °C in days; *X*_3_ = amount of rainfall (*R*) from June to November, inclusive, in terms of *R/*(*R + a*) where a = 25.4 if *R* is in mm and *a* = 1 if R is in inches; *X*_4_ = monthly mean daily maximum temperature (°C) of the warmest month; *X*_5_ = winter factor expressed in terms of (0 °C—*X*_*1*_)*R*_Jan_ where *R*_Jan_ represents the rainfall in January (mm); *X*_6_ = mean maximum snow depth (*S*) in terms of *S*/(*S* + *a*) where *a* = 25.4 if *S* is in mm and *a* + 1 if *S* is in inches; and *X*_7_ = maximum wind gust (km·h^−1^) in 30 years.

The suitability index is a value between 0 and 100 that indicates relative plant hardiness. For mapping purposes, the index values are categorized into zones and subzones based on 10- and 5-unit index intervals, respectively. For example, index values ≥ 20 and < 30 are classified as Zone 2, which is further broken down into Zone 2a (for values ≥ 20 and < 25) and Zone 2b (for values ≥ 25 and < 30). In the original (1930–1960) work, Ouellet and Sherk resolved this equation at 640 weather stations across Canada and developed (via hand interpolation) separate hardiness zone maps for eastern and western Canada. Though beyond the scope of the current work, a detailed description and assessment of the original hardiness models can be found in previous studies^5,8^.

The USDA plant hardiness index is calculated by averaging annual extreme minimum temperature (X_min_) values (i.e., the coldest winter day each year) over a period of interest (i.e., 1991–2020 in this case)^11^. Final maps are classified into zones using 5.6 °C bins, which are further divided into subzones that span 2.8 °C (USDA 2024). A total of 13 zones (26 subzones) cover the range of extreme minimum temperatures in the United States, including Alaska, Hawaii, and Peurto Rico; only 9 of these zones are found in Canada.

### Climate data and models

Climate station data covering the 1991–2020 period for variables and time steps of interest (i.e., monthly and daily minimum and maximum temperatures, monthly precipitation, and maximum monthly snow depth) were obtained from US NOAA, which includes archives of Canadian climate data from Environment and Climate Change Canada (ECCC)^28,29^. Three variables (X_2_, X_6_, and X_min_) employed station data from Canada and the United States (as required for other applications), while the remaining variables employed only Canadian data. All grids were masked to the Canadian land extent prior to calculating hardiness indices. We were unable to obtain sufficient wind and January rainfall data for the 1991–2020 period and thus utilized data from the 1981–2010 period for these variables. Though imperfect, this solution should not greatly impact the updated hardiness estimates because of: (1) large overlap between the time periods, (2) limited impact of these variables on the final hardiness index value^8^, and (3) modest trends in wind speed and rainfall over the past several decades^30^. Note that Ouellet and Sherk also had difficulty with the wind speed variable and used maps to estimate these values for their original interpolation. Only station-years with less than 20 missing values and stations with at least 10 years of data over the 1991–2020 period were included in the models. The number of climate stations available for use in this modelling effort varied across input variables, ranging from approximately 243 stations for the maximum wind model (covering Canada-only) to 9272 stations for the frost free period model (covering Canada and US) (Table 1). Variables requiring pre-processing (i.e., X_2_, X_3_, X_5_, X_6_) were calculated at each station using daily or monthly station data prior to the interpolation process described below.

**Table 1:** Model diagnostics for climate variables used in calculating the updated plant hardiness zones for the 1991–2020 period. The first seven variables were used to calculate the Canadian hardiness index; the final variable was used to calculate the USDA hardiness index.

Climate variable name	Variable ID	Units	*N* stations	Surface mean	Cross-val. MAE	Cross-val. ME	Signal: *N*
Mean Min Temp of Coldest Month	X_1_	°C	1786	− 15.02	0.89	0.00	0.34
Frost Free Period	X_2_	days	9272^a^	169.33	13.50	− 0.05	0.25
Rainfall, June–November	X_3_	mm	1879	71.44	8.88	− 0.54	0.33
Mean Max Temp of Warmest Month	X_4_	°C	1797	23.22	0.62	0.01	0.37
Rainfall, January (used in Winter Factor calculation)	X_5_	mm	1836	68.09	11.20	− 0.73	0.31
Maximum Snow Depth	X_6_	mm	8147^a^	413.59	104.00	− 6.78	0.26
Maximum Wind Gust	X_7_	km·h^−1^	243	113.73	24.90	0.00	0.60
Extreme minimum temperature	X_min_	°C	9084^a^	− 20.02	1.43	− 0.01	0.29

^a^Indicates models with spatial extent covering Canada and the United States; remaining models cover Canada only.

We used the thin plate smoothing spline-based ANUSPLIN package^31^ to develop—from the climate station data described above—spatially continuous surfaces for each of the variables used in the plant hardiness calculation. When resolved with the requisite independent variables such as latitude, longitude and elevation using a Digital Elevation Model (DEM), the result is a grid-based map (at the spatial resolution of the DEM). ANUSPLIN is a well-known suite of FORTRAN programs that calculates and optimizes multivariate (nonparametric) thin plate smoothing splines fitted to data sets with up to several thousand observations. ANUSPLIN has been widely and successfully applied to climate data around the world^32^, including North American applications^20,33^.

A general representation for a (partial) thin plate spline of *n* data values *z*_*i*_ at positions *x*_*i*_ is given by Hutchinson^34^:

2 $$z_{i} = f(x_i) + \sum\limits_{j=1}^{p} \beta_{j} \varphi_j\, (x_i) + \varepsilon_i \quad (i=1,\ldots,n;\,\, j=1,\ldots,p)$$

where *f* is an unknown smooth function to be estimated and the *β*_*j*_ are the *p* parameters of a linear sub-model. The *x*_*i*_ typically represent longitude, latitude and elevation as used in this application but are not restricted to these (e.g. aspect and large water bodies could be included as independent variables; see^35^. See recent applications^20,36^ for further background on both thin plate smoothing splines and ANUSPLIN in general. ANUSPLIN datasets contribute to temperature-precipitation simulations for Canada^37^ and to bias correction methods for high-resolution daily temperature simulations^38^.

ANUSPLIN provides several diagnostics to assess model quality. Here we report cross-validated mean error (ME) and mean absolute error (MAE) to assess model bias and accuracy, respectively. We further report the signal to number-of-data-points ratio (signal: N) for each model, which typically ranges between approximately 0.25 and 0.75—values outside this range indicate significant data errors, insufficient data, and/or an exact fit through the climate station data^39^, which ultimately results in a poor model away from the stations.

Each spatial model was resolved at a 60 arc second resolution (approximately 2 km) using a Digital Elevation Model (DEM) of Canada^40^. As noted, the DEM provides a regular grid of the independent latitude, longitude and elevation variables required to resolve the mathematical model. A computer script was written to combine the variables to generate the hardiness index described above. Results were visualized and mapped using ArcGIS Pro software^41^.

### Map comparisons

Difference maps were generated to examine shifts in hardiness index values between the current map and maps for both the 1981–2010 and the 1961–1990 periods. Frequency tables of hardiness values were also generated to further explore the changes across these time periods. In order to examine which climate variables were driving changes in the Canadian hardiness index over time, we calculated a difference map for each climate variable and time comparison of interest and then expressed these differences in terms of hardiness index values by multiplying by the corresponding regression coefficient in Eq. (1). Spatial patterns in the major drivers of change were summarized by mapping the climate variable that effected the largest absolute change in the hardiness index for each pixel across the study area. All map comparisons were carried out using the Terra package^42^ in R.

There were a number of inconsistencies between the previous hardiness maps and the current effort, which made comparison across products a challenge. These included different versions of the ANUSPLIN software, changes in the underlying climate station network, and changes to the associated workflows. Furthermore, when developing the updated map, we identified a small error in the calculation of the average minimum temperature of the coldest month (X1) for the previous 1981–2010 map^9^, which produced somewhat optimistic plant hardiness ratings (by approximately half a zone), primarily in the southern Prairies region of Canada. Consequently, for the comparisons presented here, we employed updated and corrected versions of the previous maps, allowing for more consistent comparisons over time.

## Results and discussion

### Climate models

Spatial models of the seven climate variables used to calculate the Canadian plant hardiness index exhibited error metrics and associated model diagnostics (Table 1) that were comparable to those reported in other studies^20,32^. Cross-validated MAEs were 0.89 °C and 0.62 °C for the monthly min and max temperature variables (X_1_ and X_4_), respectively. Frost free period (X_2_), another temperature-driven variable, exhibited an MAE of 13.50 days—or approximately 8% of the surface mean (i.e., average over the entire modelled area). Mean absolute errors associated with the rainfall-related variables, X_3_ and X_5_, were 8.88 mm (12%) and 11.20 mm (16%), respectively. Errors associated with maximum snow depth (104 mm or 25%) and maximum wind gust (24.9 km/h or 22%) were relatively high, reflecting the high spatial variability, significant measurement error, and limited number of reporting stations for these variables across Canada. The USDA extreme minimum temperature model exhibited an MAE of 1.41 °C. This is somewhat higher than the errors associated with the monthly temperature variables reported above, reflecting the larger absolute values and higher spatial variation associated with the daily climate extremes used for this zonation approach. Mean error (i.e., bias) was small for all variables, though there was some evidence of modest negative bias (i.e., − 6.78 mm or -2%) in the snow depth model. The signal: N diagnostic ranged from 0.25 to 0.60—indicating robust models that fit data patterns without forcing exact interpolations through station values^39^. Maps of the seven climate variables used to calculate the Canadian hardiness index are provided in supplementary material (Figs. S1–S7).

### Updated hardiness zone maps

**Fig. 1:**
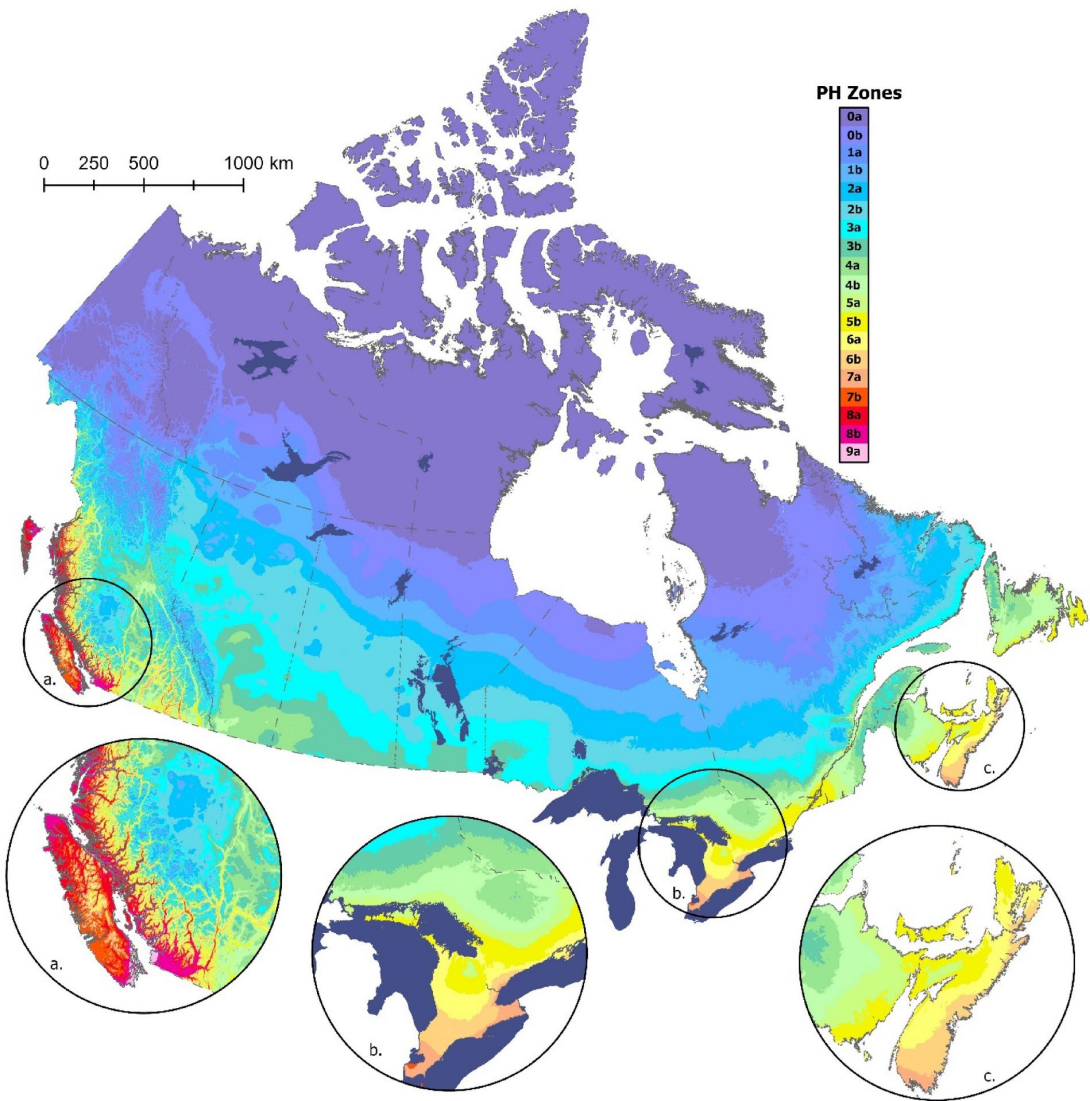
The Canadian plant hardiness zone map for the 1991–2020 climate period, with insets showing greater detail for: (**a**) southwestern British Columbia, (**b**) southern Ontario, and (**c**) the Maritime provinces of Nova Scotia, New Brunswick, and Prince Edward Island. Map produced using ArcGIS Pro^41^.

Figure 1 shows the updated Canadian plant hardiness zone map for the 1991–2020 climate normal period. Spatial patterns in plant hardiness generally follow those of previous versions of the map, with highest values in the coastal and interior regions of southern British Columbia, southern Ontario, and the southern Maritimes region (Fig. 1a–c). Low hardiness values are found in the far north and in mountainous regions, such as the Canadian Rockies along the border of British Columbia and Alberta. More subtle elevation effects can be seen throughout the country, including Duck Mountain near the Manitoba-Saskatchewan border, the Algonquin Highlands in southeastern Ontario near the Quebec border, and in the Laurentian Mountains north of Quebec City (Fig. 1).

The most common plant hardiness subzone under the Canadian system is 0a (50.3%), which covers the far northern portion of the country and is not suitable for most horticultural plants (Table 2; Fig. 1). Areal coverage by the remaining subzones decreases steadily with increasing hardiness rating. The least common hardiness zone in Canada is 9a (0.01%), which is found only at the southern end of Vancouver Island (in and around the city of Victoria) and the southwestern-most portion of the British Columbia mainland (in and around the city of Vancouver) (Fig. 1a).

**Table 2:** Count and percentage of pixels in each Canadian plant hardiness class for each of three time periods 1991–2020, 1981–2010, and 1961–1990.

PH class	1991–2020		1981–2010		1961–1990	
	Count	Percent	Count	Percent	Count	Percent
0a	3,101,645	50.30	3,199,119	51.88	3,671,560	59.55
0b	532,270	8.63	552,292	8.96	451,009	7.31
1a	403,679	6.55	436,381	7.08	407,963	6.62
1b	373,485	6.06	358,321	5.81	433,954	7.04
2a	362,460	5.88	398,613	6.46	322,080	5.22
2b	423,738	6.87	372,939	6.05	264,780	4.29
3a	310,910	5.04	276,247	4.48	205,232	3.33
3b	205,898	3.34	191,809	3.11	109,645	1.78
4a	135,863	2.20	113,312	1.84	73,359	1.19
4b	90,522	1.47	68,251	1.11	52,247	0.85
5a	55,169	0.89	51,948	0.84	54,588	0.89
5b	53,250	0.86	48,009	0.78	39,644	0.64
6a	34,330	0.56	29,574	0.48	22,177	0.36
6b	24,600	0.40	20,358	0.33	18,212	0.30
7a	17,617	0.29	14,617	0.24	14,540	0.24
7b	12,052	0.20	14,399	0.23	15,226	0.25
8a	17,572	0.28	15,098	0.24	9564	0.16
8b	10,207	0.17	4535	0.07	229	0.00
9a	742	0.01	187	0.00	0	0.00

**Fig. 2:**
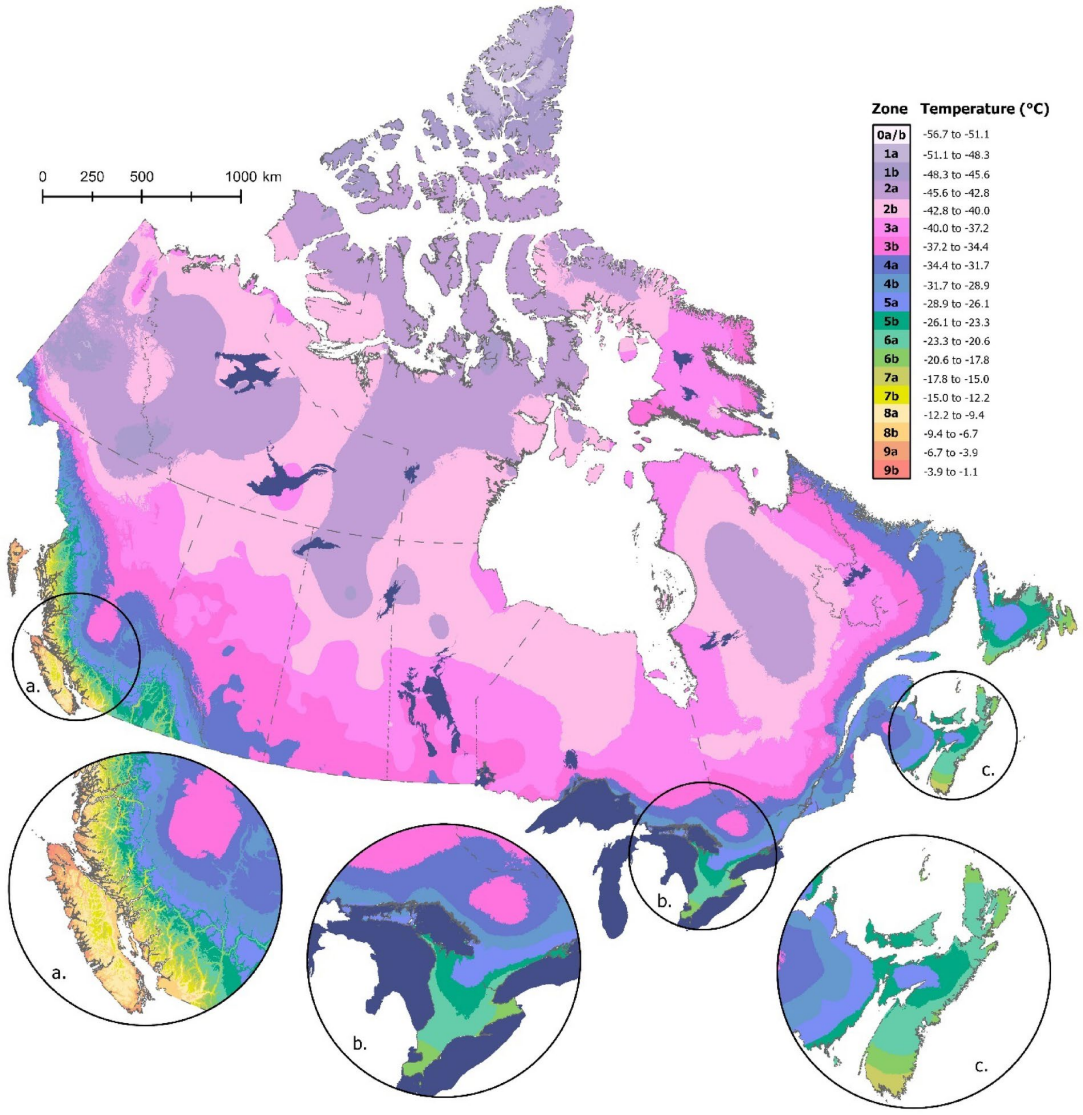
The USDA plant hardiness zone map for Canada for the 1991–2020 climate period, with insets showing greater detail for: (**a**) southwestern British Columbia, (**b**) southern Ontario, and (**c**) the Maritime provinces of Nova Scotia, New Brunswick, and Prince Edward Island. See Table 3 for details on the extreme minimum temperature ranges associated with each hardiness zone. Map produced using ArcGIS Pro^41^.

Spatial patterns in plant hardiness associated with the USDA-based map (Fig. 2) were generally similar to those reported for the Canadian approach, with variation related primarily to latitude, elevation, and coastal proximity (Fig. 2a–c). The most common USDA hardiness class in Canada is Zone 2 (55.2%), which is widely distributed across the northern portion of the country (Table 3). Areal coverage of the remaining classes declines with increasing hardiness rating. Subzone 9b, located almost entirely at the northern tip of Vancouver Island, British Columbia (Fig. 2a), is the least common USDA hardiness class in Canada—though there are four zones in the United States (i.e., Zones 10–13) that are not present in Canada. Despite the general similarities between the two products, previous work has found that the extreme minimum temperature zones cover multiple Canadian zones, hence there is no simple way to convert between the Canadian and US systems^13^.

**Table 3:** Count and percentage of pixels in each USDA plant hardiness class for each of three time periods 1991–2020, 1981–2010, and 1961–1990.

PH Class	1991–2020		1981–2010		1961–1990	
	Count	Percent	Count	Percent	Count	Percent
1a	157,216	2.55	265,988	4.31	549,182	8.91
1b	376,352	6.10	484,338	7.85	1,580,376	25.63
2a	1,686,644	27.35	1,873,098	30.38	1,286,562	20.86
2b	1,716,293	27.83	1,676,162	27.18	1,347,684	21.85
3a	992,825	16.10	844,290	13.69	601,188	9.75
3b	492,728	7.99	397,992	6.45	284,064	4.61
4a	269,307	4.37	227,661	3.69	168,698	2.74
4b	144,746	2.35	112,276	1.82	104,916	1.70
5a	105,520	1.71	94,267	1.53	86,819	1.41
5b	78,293	1.27	67,101	1.09	51,905	0.84
6a	56,476	0.92	47,319	0.77	40,113	0.65
6b	31,360	0.51	24,625	0.40	18,068	0.29
7a	13,272	0.22	9344	0.15	7950	0.13
7b	8753	0.14	8218	0.13	8785	0.14
8a	12,442	0.20	11,933	0.19	13,347	0.22
8b	13,945	0.23	15,256	0.25	14,475	0.23
9a	9839	0.16	6588	0.11	2385	0.04
9b	506	0.01	61	0.00	0	0.00

### Changes in hardiness zones

**Fig. 3:**
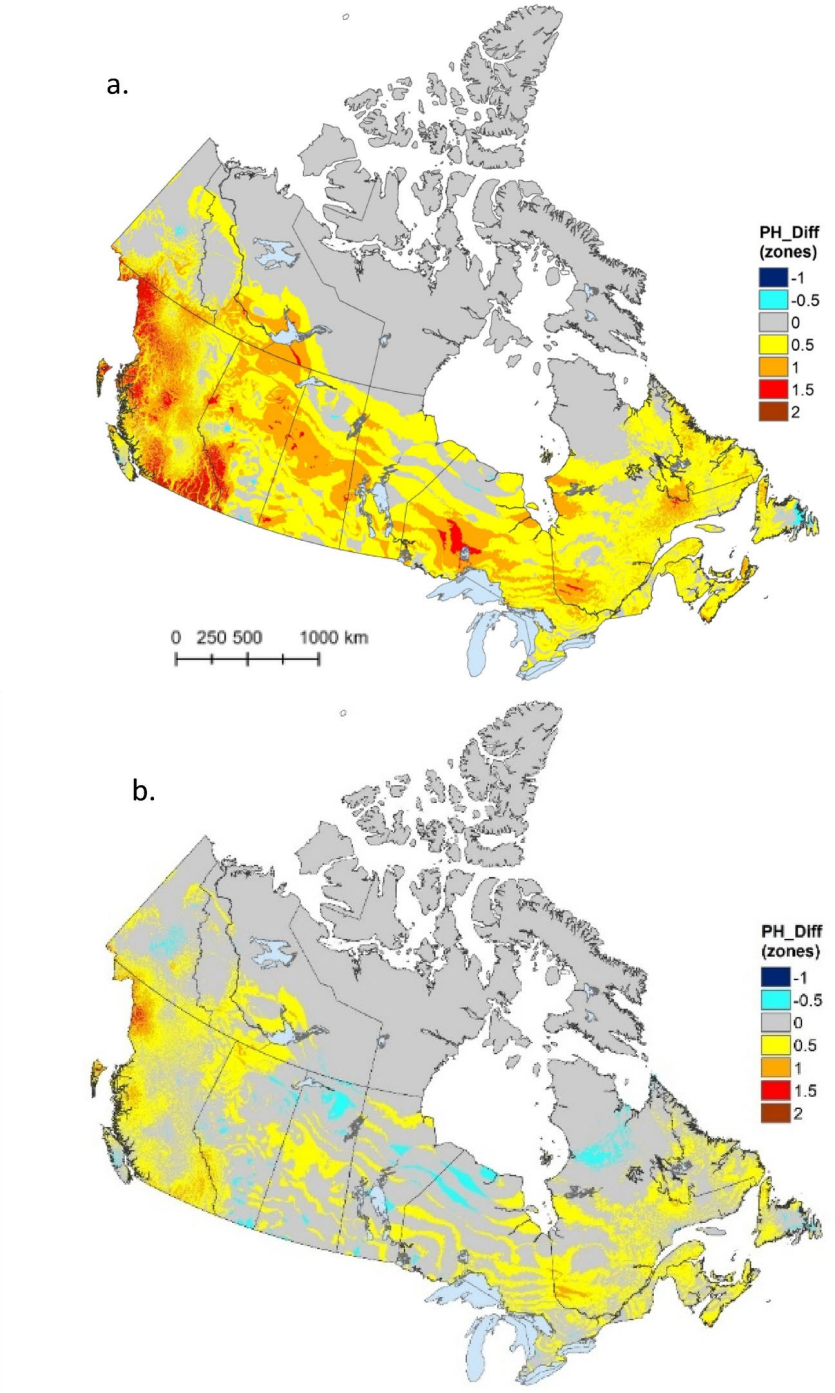
Change in Canadian plant hardiness zones for (**a**) 1991–2020 period minus 1961–1990 period and (**b**) 1991–2020 period minus 1981–2010 period. Changes are expressed as number of zones. Map produced using ArcGIS Pro^41^.

Relative to the 1961–1990 period, much of southern Canada has experienced an increase in Canadian hardiness zone ratings, with the largest increases (up to 2 full zones) happening in western Canada—particularly in southern and northwestern British Columbia (Fig. 3a). Much of the far northern portion of the country did not change as the hardiness index is not calibrated for the extremely cold conditions in this region and thus did not reflect warming that may have occurred there. Of the pixels that exhibited a change in hardiness rating, the vast majority were in a positive/upward direction (99.6%; Table 4); however, hardiness ratings declined by half a zone in a few scattered locations, including northeastern Newfoundland, the western shore of Vancouver Island, and several small pockets across the Prairie provinces (Fig. 3a). Similar, though less dramatic, patterns of change were apparent when the current hardiness map was compared to the map developed for the previous climate normal period of 1981–2010 (Fig. 3b; Table 4). More modest differences were expected in this case, given that the climate normal periods being compared overlap by 20 years. Nevertheless, 92% of pixels exhibiting change were in a positive direction (Table 4).

**Table 4:** Count and percentage of pixels in each change class (expressed as change in number of hardiness zones) for comparisons of the Canadian plant hardiness index between two time periods: (1) 1991–2020 minus 1961–1990 and (2) 1991–2020 minus 1981–2010.

Change (# of zones)	1991–2020 minus 1961–1990		1991–2020 minus 1981–2010	
	Count	Percent	Count	Percent
− 1	118	0.00	3	0.00
− 0.5	9379	0.15	100,714	1.63
0	3,482,720	56.48	4,923,257	79.85
0.5	1,766,072	28.64	1,074,726	17.43
1	797,285	12.93	64,164	1.04
1.5	101,955	1.65	3145	0.05
2	8416	0.14	0	0.00

**Fig. 4:**
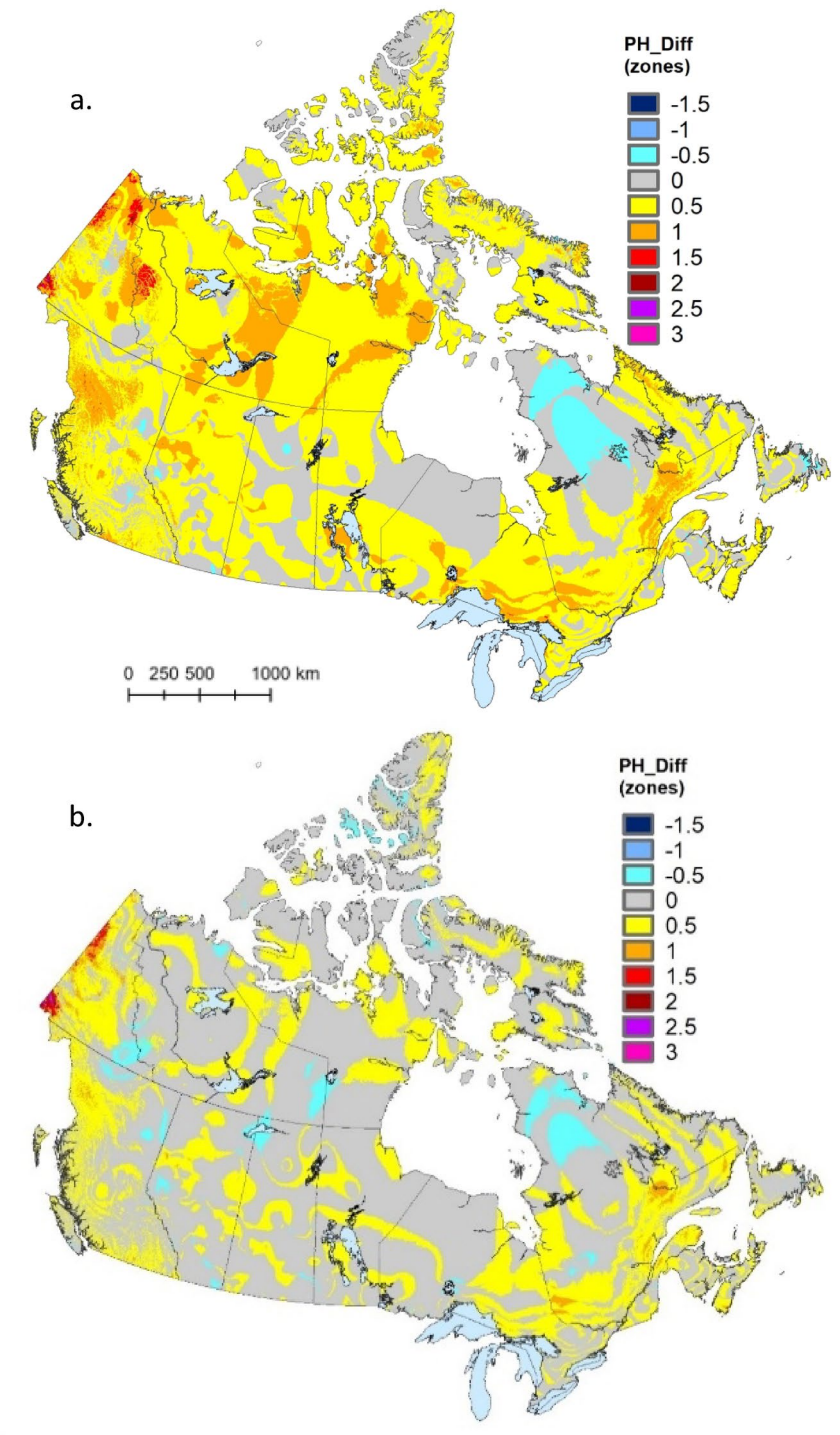
Change in USDA (i.e., extreme minimum temperature) plant hardiness zones for (**a**) 1991–2020 period minus 1961–1990 period and (**b**) 1991–2020 period minus 1981–2010 period. Changes are expressed as number of zones. Map produced using ArcGIS Pro^41^.

Comparable shifts were apparent when comparing USDA hardiness zones across time periods (Fig. 4). Again, when comparing the current map to that for the 1961–1990 period, the vast majority of shifts (96.9%) were toward warmer zones (Fig. 4a; Table 5). Though less dramatic, shifts were also predominantly (89.2%) toward warmer conditions when comparing the current USDA hardiness map to that for the 1981–2010 period (Fig. 4b; Table 5). For both comparisons, the largest increases were in northwestern Canada, including the Yukon and Northwest Territories, while a persistent half zone decline was apparent in northern Québec.

**Table 5:** Count and percentage of pixels in each change class (expressed as change in number of hardiness zones) for comparisons of the USDA plant hardiness index between two time periods: (1) 1991–2020 minus 1961–1990 and (2) 1991–2020 minus 1981–2010.

Change (# of zones)	1991–2020 minus 1961–1990		1991–2020 minus 1981–2010	
	Count	Percent	Count	Percent
− 2	0	0.00	3	0.00
− 1.5	1	0.00	15	0.00
− 1	50	0.00	561	0.01
− 0.5	139,992	2.27	214,767	3.48
0	1,607,919	26.07	4,178,047	67.75
0.5	3,666,253	59.45	1,707,815	27.69
1	723,379	11.73	51,565	0.84
1.5	27,001	0.44	9746	0.16
2	1645	0.03	2547	0.04
2.5	226	0.00	1164	0.02
3	51	0.00	229	0.00
3.5	0	0.00	58	0.00

Further evidence of change is apparent in the hardiness class frequencies associated with each time period (Tables 2 and 3). Relative to the earlier products, the current maps (both Canadian and USDA) generally have a lower percentage of pixels in the lower hardiness classes and a higher percentage in the higher classes. This transition is particularly evident in the highest hardiness zones; for example, Canadian Zone 9a did not exist on the 1961–1990 map, was present in small numbers on the 1981–2010 map, and has now spread to include much of the area around Vancouver and Victoria in extreme southwestern British Columbia (Fig. 1; Table 2). Similarly, USDA Zone 9b has steadily expanded over time and now covers much of the northern tip of Vancouver Island (Fig. 2; Table 3).

It is noteworthy that hardiness zone declines were reported in all change summaries presented here—albeit much less commonly than hardiness zone increases. We note several qualifications in relation to these (perhaps unexpected) results. First, climate change is not expected to progress evenly across Canada, and these results may—at least in part—reflect variation in the magnitude (and even direction) of climate change across climate variables and locations^43^. Furthermore, climate variables that are driven by event-based phenomena (e.g., start/end of the growing season) or that report climate extremes (e.g., X_min_) may be sensitive to outlier/random events and thus variable in both magnitude and direction at any given location^44^. Finally, we note that the climate station network is very limited in some portions of Canada, making map interpolations, such as those undertaken here, sensitive to subtle changes in the underlying station network. This may explain the modest decline in the USDA hardiness rating in northern Québec (Fig. 4)—a region with very few climate stations.

Shifts toward higher hardiness ratings over time were previously reported from updates to hardiness maps in both Canada^8,9^ and the United States^11^. Similar to the spatial patterns reported here (Fig. 3), Canadian hardiness ratings have consistently shown larger shifts in the western portion of the country^8,9^, likely reflecting steeper warming trends in this region over the past half century^27^. East-west variation in shifts of the USDA-based hardiness index was less evident (Fig. 4), though there were several areas in the far northwest (i.e., Yukon and Northwest Territories) where increases of greater than 1.5 zones occurred.

Shifts in hardiness zones are expected to provide new horticultural opportunities over time. For example, the harvested area of grapes in Ontario has increased by approximately 25% over the 1990 to 2020 period^45^, with production expanding to the north of traditional grape growing areas in the extreme southern portion of the province^46^. Similar expansions have occurred in other wine-producing regions of Canada, including southern Québec^47^ and the Okanagan Valley of British Columbia^48^—in both cases, shifts have been attributed to past and projected future climate warming^49–51^. While hardiness-related shifts have started for some crops in some areas, large-scale changes are not yet apparent. This likely reflects that significant portions of the country have experienced modest (or no) change in hardiness rating (Tables 4 and 5). Furthermore, producers may be reticent to shift to less hardy crops when there are still significant risks associated with extreme climate events such as extreme winter temperatures and late spring/early fall frosts^9^.

### Change attribution

**Fig. 5:**
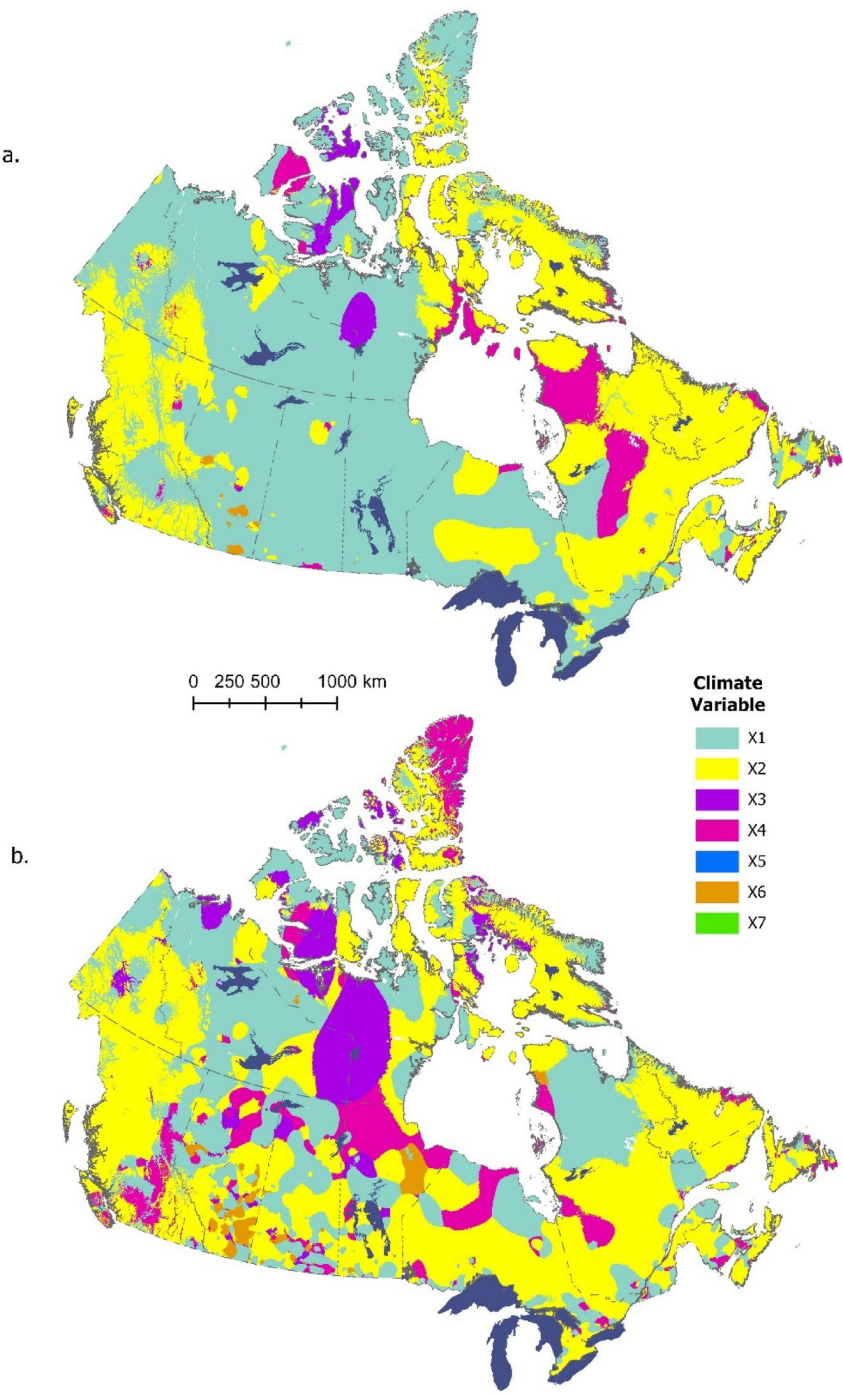
Climate variable contributing the greatest amount to change in plant hardiness index at locations across Canada for (**a**) 1991–2020 period versus 1961–1990 period and (**b**) 1991–2020 period versus 1981–2010 period. Climate variables include (see text for full definitions): X1 = Minimum temperature of coldest month; X2 = Growing Season Length; X3 = Rain Index; X4 = Maximum temperature of hottest month; X5 = Winter Factor; X6 = Snow Depth; and X7 = Max Wind Gust. Map produced using ArcGIS Pro^41^.

Changes in Canadian hardiness index between 1961 and 1990 and 1991–2020 were driven primarily by changes in temperature-related variables, including average minimum temperature of the coldest month (X_1_), frost free period (X_2_), and average maximum temperature of the hottest month (X_4_) (Fig. 5a). These three variables were the primary drivers of change across 97% of the pixels in the study area. Other climatic drivers of note include the rain index (X_3_), which was the primary driver of change across a relatively small area in the far north, and maximum snow depth, which drove change in scattered pockets across southern Alberta. Climatic drivers of change between the current and 1981–2010 period exhibited generally similar patterns to those described above, with temperature-related variables driving change across 90% of pixels (Fig. 5b). In this case, the rain index drove change across a larger portion of the far north, while maximum snow depth drove change in pockets across the entire Prairie region. The more spatially variable nature of the patterns in Fig. 5b (relative to Fig. 5a) likely reflects the reduced change signal associated with the heavily overlapping time periods being compared.

We note a number of caveats to these findings. First, while the maps in Fig. 5 identify a primary climate variable that drives change at each location, the magnitude of change may not translate into a shift in hardiness zone rating, depending on where the updated raw hardiness value falls relative to the cut-off values for each hardiness class. A good example of this is the far north, where the hardiness zone classification (0a) did not change (see Fig. 3) despite changes in several temperature-related variables (Fig. 5). Further, the change attribution maps show the single climate variable that brings about the largest change in the raw plant hardiness index at a given location, but the other climate variables also contribute to the final updated value—and in some cases may even counteract the influence of the main driver. Despite these qualifiers, we feel there is value in presenting an overall picture of change attribution as provided by the maps in Fig. 5.

These findings are generally supported by previous work examining trends in Canadian climate. Several studies have reported positive trends in temperature-related variables (including frost free period, and minimum and maximum temperatures) over the past 50–100 years in Canada^27,44,52–54^. A consistent finding across these studies is for greater warming in western Canada, which generally supports the patterns of change in hardiness ratings reported here (Fig. 3). Furthermore, these studies typically report moderate and spatially variable changes in precipitation, which is consistent with the modest role attributed to the rainfall variable (X_3_) in the change attribution analysis presented above. Previous work has identified the temperature-related variables (X_1_, X_2_, and X_4_) as key drivers of the calculated plant hardiness rating at a given location^6,8^; thus, their importance in driving changes in hardiness ratings reflects both significant trends over time and their weighting in the regression model used to calculate the hardiness index.

### Caveats and future work

There are a number of challenges to comparing plant hardiness maps over time. The software employed here for spatial interpolation (i.e., ANUSPLIN) has undergone several upgrades over the past decade, which has implications for the consistency of the underlying climate grids that drive the hardiness calculation. Furthermore, climate station networks in Canada have been declining in size for several decades^36^, which further complicates comparisons across periods. Consequently, we elected to make comparisons using the most recent version of the ANUSPLIN software and a relatively stable set of climate stations. This approach allowed for consistency in comparisons across time periods, though we recognize that the products being compared may differ slightly from the officially released products at the time. We also note that the spatial resolution of the hardiness maps presented here (approximately 2-km) may result in some inaccuracies in highly variable landscapes, such as mountainous and coastal regions. However, this resolution reflects the meso-scale nature of the underlying climate models, which is ultimately dictated by the modest density of climate stations in much of Canada^20^.

We recognize several potential projects related to plant hardiness in Canada. One area of future work is the development of a heat zone map, similar to that developed in the United States^14^, which identifies areas with different levels of potential heat damage to plants. Although the Canadian plant hardiness model incorporates maximum temperature (i.e., variable X_4_), it does not explicitly identify heat-related risks. Another topic of interest is the development of maps showing projected plant hardiness ratings under climate change (see Qian et al.^55^ for a recent example using the USDA approach). While such a product would have value for anticipating future planting opportunities at locations of interest, we have found it challenging to generate future climate grids for several of the required Canadian plant hardiness variables, including winter factor (X_5_), snow index (X_6_), and maximum wind gust (X_7_). In lieu of future hardiness zone projections, current and future distribution models have been developed for several thousand native and horticultural plant species^18^. This work is ongoing and would benefit from expanded collaboration between the nursery industry and scientific modeling groups such as ours to establish a plant survival monitoring network at locations across Canada. Such an effort would support improvements to the existing hardiness zone system as well as the development of species-specific distribution models. Finally, the influence of the length of the climate period used to calculate the hardiness zone index should be explored; weather events and anticipated rapid climate shifts may necessitate a shorter reporting period than that currently employed (i.e., 30 years in the current work).

As climate continues to change, it is expected that the Canadian plant hardiness system will eventually be pushed beyond the climatic limits for which it was originally calibrated. For example, when hardiness index values greater than 100 start to appear, this may necessitate the development of new zones—or even a new hardiness system altogether. However, for the current update, the maximum index value was 93.5 (i.e., zone 9a), which indicates there is still room for some upward expansion within the current system.

One challenge with developing a single hardiness zone system for a country as large as Canada is that there is, inevitably, regional variation in how well the zones capture hardiness constraints on plants across the country. For example, anecdotal evidence suggests that extreme winter temperatures may be the primary driver of plant range limits in the Prairie region, growing season length may be more important under the milder conditions of southeastern Canada, while wind may be a key driver in cool coastal regions such as Newfoundland. While our focus here, and in previous efforts, has been to faithfully reproduce the established Canadian and USDA hardiness zone models, future efforts may explore the development of a new system that addresses these regional challenges. Such a product could incorporate elements of both the Canadian and USDA systems, new and more comprehensive plant survival data as noted above, and employ modern, machine learning tools to guide zone delineation.

## Concluding comments

Here we report on an effort to update plant hardiness zone maps for Canada, using climate data for the 1991–2020 period. Relative to previous hardiness maps, the current map shows a shift toward higher (i.e., warmer) zones at many locations across Canada, with the largest changes occurring in the western and northwestern portions of the country. These shifts, driven primarily by temperature increases, indicate the potential for new planting opportunities; though modest (or no) changes in some areas, combined with ongoing risks from extreme climate phenomena, such as droughts and false spring events, will likely slow the transition to new plant/crop species across Canada. The updated maps, along with species-specific climate habitat models, are available at http://planthardiness.gc.ca.

## Electronic supplementary material

Below is the link to the electronic supplementary material.

Supplementary Material 1

## Data Availability

All spatial data for the study will be openly accessible on the Canadian Federal Geospatial Platform at https://ftp.maps.canada.ca/pub/nrcan_rncan/Climate-archives_Archives-climatologiques.
