# Supplementary material for: Updated plant hardiness zones for Canada and assessment of change over time
Source: Sci Rep. 2025 Jul 2;15:22774. doi: 10.1038/s41598-025-00931-5 (PMC12216870; doi:10.1038/s41598-025-00931-5)

**Supplementary Information**

Updated plant hardiness zones for Canada and assessment of change over time

**Figure S1**. Map of monthly mean daily minimum temperature (°C) of the coldest month for Canada – variable X1 in the Canadian plant hardiness calculation. Map produced using ArcGIS Pro Version 3.0 (<https://www.esri.com/en-us/arcgis/products/arcgis-pro/overview>).


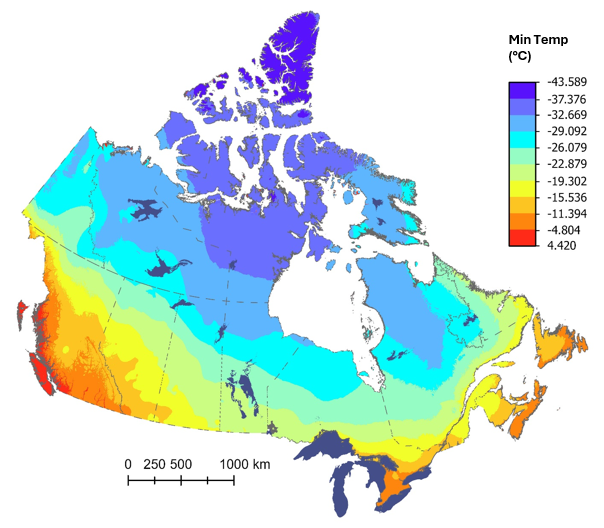


**Figure S2**. Map of frost free period (number of days) for Canada – variable X2 in the Canadian plant hardiness calculation. Map produced using ArcGIS Pro Version 3.0 (<https://www.esri.com/en-us/arcgis/products/arcgis-pro/overview>).


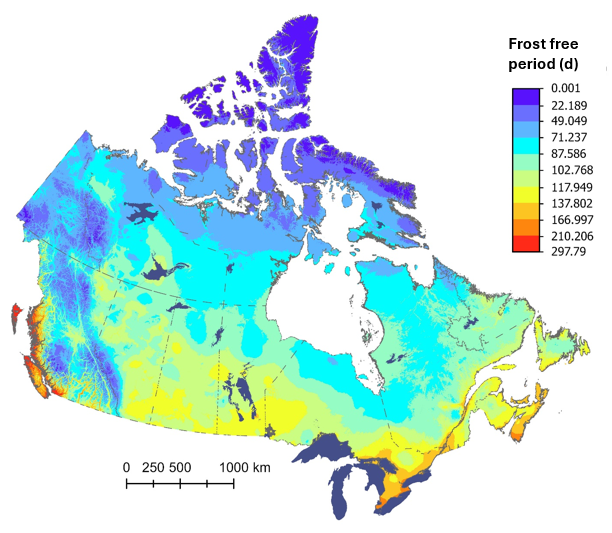


**Figure S3**. Map of rain index for Canada – variable X3 in the Canadian plant hardiness calculation. This variable is calculated as R/(R+a), where R is the amount of rainfall (in mm) from June to November, inclusive, and a=25.4. Map produced using ArcGIS Pro Version 3.0 (<https://www.esri.com/en-us/arcgis/products/arcgis-pro/overview>).


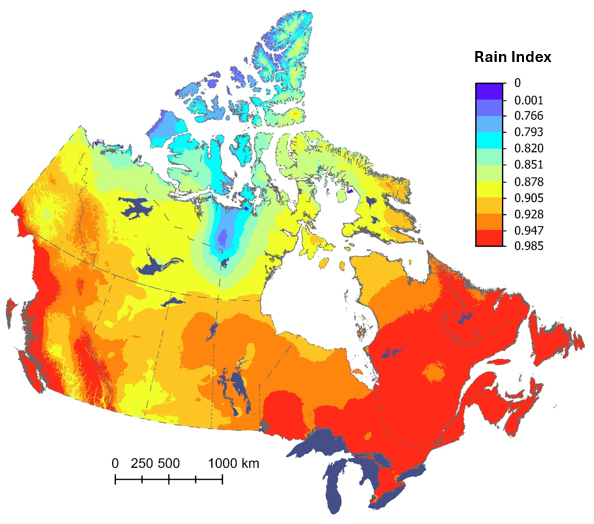


**Figure S4**. Map of monthly mean daily maximum temperature (^o^C) of the warmest month for Canada – variable X4 in the Canadian plant hardiness calculation. Map produced using ArcGIS Pro Version 3.0 (<https://www.esri.com/en-us/arcgis/products/arcgis-pro/overview>).


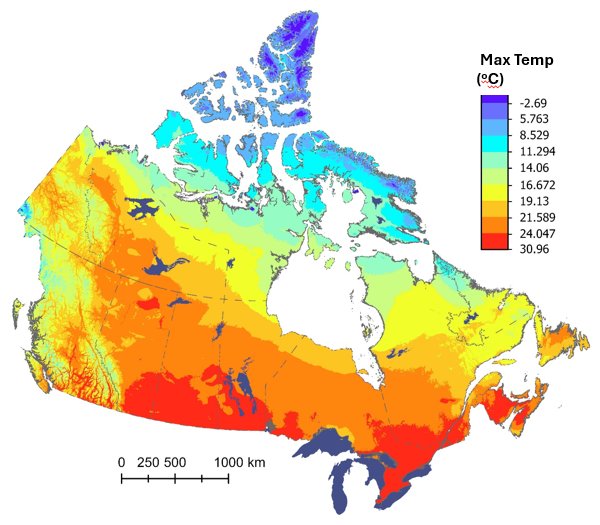


**Figure S5**. Map of winter factor for Canada – variable X5 in the Canadian plant hardiness calculation. This variable is calculated as (0^o^C – X1)·R_Jan_, where R_Jan_ is rainfall in January (mm) and X1 is the average daily minimum temperature of the coldest month (see Figure S1). Map produced using ArcGIS Pro Version 3.0 (<https://www.esri.com/en-us/arcgis/products/arcgis-pro/overview>).


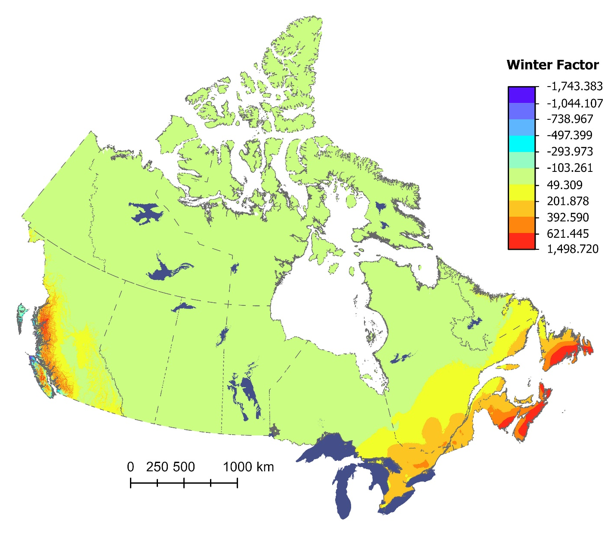


**Figure S6**. Map of mean maximum snow depth factor for Canada – variable X6 in the Canadian plant hardiness calculation. This variable is calculated as (S/(S+a)), where S is mean maximum snow depth (mm) and a = 25.4. Map produced using ArcGIS Pro Version 3.0 (<https://www.esri.com/en-us/arcgis/products/arcgis-pro/overview>).


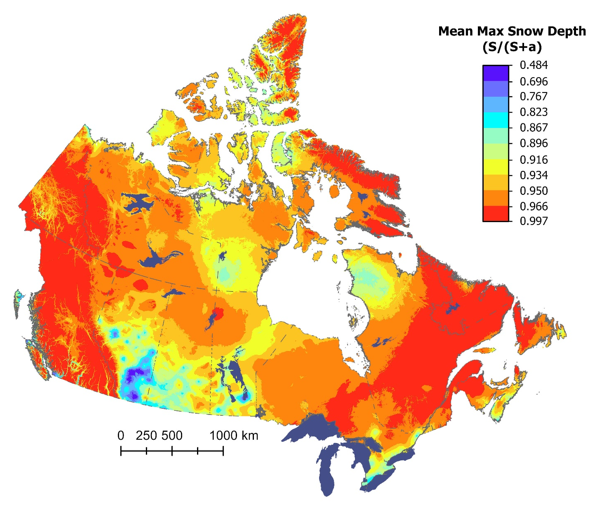


**Figure S7**. Map of maximum wind gust (km·h^-1^) for Canada – variable X7 in the Canadian plant hardiness calculation. Map produced using ArcGIS Pro Version 3.0 (<https://www.esri.com/en-us/arcgis/products/arcgis-pro/overview>).


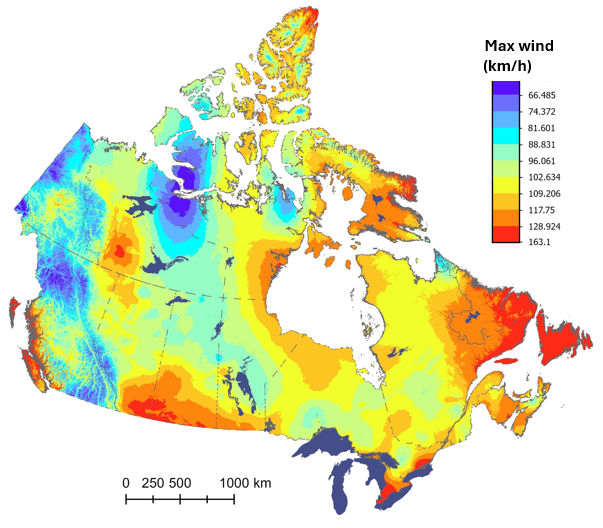

Supplement: Supplementary file 1 — Supplementary Material 1 [file 41598_2025_931_MOESM1_ESM.docx]
